# Space-Time Analysis of Testicular Cancer Clusters Using Residential Histories: A Case-Control Study in Denmark

**DOI:** 10.1371/journal.pone.0120285

**Published:** 2015-03-10

**Authors:** Chantel D. Sloan, Rikke B. Nordsborg, Geoffrey M. Jacquez, Ole Raaschou-Nielsen, Jaymie R. Meliker

**Affiliations:** 1 Department of Preventive Medicine, Stony Brook University, Stony Brook, New York, United States of America; 2 Department of Health Science, Brigham Young University, Provo, Utah, United States of America; 3 Danish Cancer Society Research Center, Copenhagen, Denmark; 4 BioMedware, Inc., Ann Arbor, Michigan, United States of America; 5 Department of Geography, State University of New York at Buffalo, Buffalo, New York, United States of America; 6 Program in Public Health, Stony Brook University, Stony Brook, New York, United States of America; National Cancer Center, JAPAN

## Abstract

Though the etiology is largely unknown, testicular cancer incidence has seen recent significant increases in northern Europe and throughout many Western regions. The most common cancer in males under age 40, age period cohort models have posited exposures in the *in utero* environment or in early childhood as possible causes of increased risk of testicular cancer. Some of these factors may be tied to geography through being associated with behavioral, cultural, sociodemographic or built environment characteristics. If so, this could result in detectable geographic clusters of cases that could lead to hypotheses regarding environmental targets for intervention. Given a latency period between exposure to an environmental carcinogen and testicular cancer diagnosis, mobility histories are beneficial for spatial cluster analyses. Nearest-neighbor based Q-statistics allow for the incorporation of changes in residency in spatial disease cluster detection. Using these methods, a space-time cluster analysis was conducted on a population-wide case-control population selected from the Danish Cancer Registry with mobility histories since 1971 extracted from the Danish Civil Registration System. Cases (N=3297) were diagnosed between 1991 and 2003, and two sets of controls (N=3297 for each set) matched on sex and date of birth were included in the study. We also examined spatial patterns in maternal residential history for those cases and controls born in 1971 or later (N= 589 case-control pairs). Several small clusters were detected when aligning individuals by year prior to diagnosis, age at diagnosis and calendar year of diagnosis. However, the largest of these clusters contained only 2 statistically significant individuals at their center, and were not replicated in SaTScan spatial-only analyses which are less susceptible to multiple testing bias. We found little evidence of local clusters in residential histories of testicular cancer cases in this Danish population.

## Introduction

Testicular cancer incidence in Western countries dramatically increased from the 1940’s to the 1990’s, and has become the number one cause of cancer in males under age 40 [1]. The exact reasons remain unclear, though a birth cohort effect has been shown to be an important factor [2–4]. The increase in rates was especially heavy in northern Europe. From 1943 to 2003 the incidence rates in Denmark increased from 3.4 to 10 cases per 100,000 person-years; its rates are among the highest in the world [3,5]. In recent years, these rates appear to be stabilizing [6].

Biologically, most testicular cancer can be categorized as either seminomas (germ cell) or nonseminomas. Seminomas typically occur later than nonseminomas (late 30’s to early 50’s versus younger than 30), indicating that there may be etiological differences between the two groups. Despite many potential risk factors that have been examined, the only firm determinants of testicular cancer risk remain age, family history of testicular cancer, national origin, birth year, ethnicity, and cryptochoridism (undescended testes) which is found in 10% of all cases [4,7–11]. Further evidence suggests that maternal factors, early childhood environment, and the *in utero* environment may play a role since testicular cancer usually occurs in the relatively young, but specific risk factors are as yet unidentified [12–14]. Certainly there is a latency period between exposure to carcinogens and disease manifestation, which should be taken into account when conducting an epidemiologic analysis of testicular cancer.

A spatial analysis in Britain was conducted but without finding evidence of responsible factors that were geographically-differentiated [15]. Though this study looked at testicular cancer rates over several years, they were not able to account for human mobility and used aggregate data (electoral wards) rather than individual-level data. Exposures leading to testicular cancer may occur many years before diagnosis, therefore cluster detection based on residence at diagnosis may not accurately reflect the life history associated with etiology.

Latency is a difficult barrier in investigating spatial cancer clusters, as individuals are often highly mobile and will move residences more than once over the course of their life [16–18]. Several methodologies have recently been developed that help solve this difficult statistical task, with two of the most promising being generalized additive models (GAMs) with a loess smoother [19] and Q-statistics [20–22]. Each of these methods allows for covariate adjustment and incorporates the use of time-specific geographic coordinates. Q-statistics have recently been shown to be effective at locating simulated clusters [23], and are here applied on a large testicular cancer dataset from Denmark.

By systematically examining residential histories, geographical clusters of testicular cancer may appear that can yield clues to what has driven the recent rise in incidence. Here we tested this hypothesis by undertaking a space-time analysis of residential histories of Danish testicular cancer cases and 2 sets of matched controls. This analysis is the first of its kind, in that it uses a comprehensive, nation-wide longitudinal database including all residential histories in Denmark to investigate local clusters of testicular cancer over a long time period. We also selected two independent control groups, something rarely done in spatial epidemiology, but recently shown to impact findings of spatial cluster analyses [22].

## Materials and Methods

### Study Population

The Danish Cancer Registry is a comprehensive nationwide cancer registry begun in 1943. For the purposes of this study, we selected testicular cancer cases diagnosed between January 1^st^, 1991 and December 31^st^, 2003 (n = 3,297) (Table 1). All individuals had testicular cancer as their primary cancer diagnosis with ICD-O morphology behaviour code 3 (malignant, primary site); however, preceding diagnosis of non-melanoma skin cancer was allowed. Seminoma testicular cancer was defined as ICD-O morphology codes: 9060/3, 9061/3, 9062/3 and 9063/3. The remaining cases were classified as non-seminoma. There were 1871 seminoma and 1426 non-seminoma cases. Two independent control groups were randomly drawn from the Danish Civil Registration System (CRS) and matched with cases according to age in a 1:1 ratio. More specifically, each control group contained 3,297 males born in Denmark on the same date as a cancer case (or same month if there were no alive controls born on the same day) that were cancer-free (with the exception of non-melanoma skin cancer) and living in Denmark at the time of the matching cases’ diagnosis. The use of two independent control groups is supported by recent work that suggests cluster results were not consistent across two control groups, leading to the conclusion that the spatial distribution of the control groups might be influencing findings of clusters [22]. Residential histories of cases and controls were traced using data from the CRS from 1971 to date of diagnosis. Geocoding success and accuracy were very high, with geographic coordinates assigned to 98% of the addresses (67,244 / 68,578). For those matched to the home address (88% of residences) the address point is defined within 1 meter of the front door of the house, and the precision of the geocoding is within a few meters. Another 4% were matched at the neighbor’s residence, 1% at the street level, and 5% at the municipality level. The Danish Data Protection Agency approved the study. In accordance with Danish law written consent was not obtained as the study was entirely register-based and did not involve biological samples from, or contact with study participants. IRB approval of work with geographic coordinates of residences was obtained through Western Institutional Review Board. Cases and controls were de-identified and assigned a random ID prior to analysis. Some protected health information (location and date of illness) were required to complete the study, but were not associated with patient identifiable information during the analysis.

**Table 1:** Characteristics of Study Population. Testicular cancer cases diagnosed 1991–2003, and age-matched controls.

	Cases	Control Group 1	Control Group 2
No. in Study population	3,297	3,297	3,297
No. of residences	22,541	22,356	22,347
No. of Seminomas	1,871	1,871	1,871
No. of residences	13,155	13,027	12,855
No. of Mothers with address histories before birth of child	591	589	591
No. of residences	1,916	1,914	1,966

We also collected residential addresses of the mothers of cases and controls from 1971 to date of birth of the case or control. This information was available for the youngest 18% of the case-control pairs. These data were used to investigate space-time local clusters of the mothers of cases and controls during pregnancy. If one of the individuals in a case-control pair had missing information on the mother’s address, the pair was excluded from the analyses. Information on mothers’ addresses existed for 589 case-control pairs in the first case-control group, and 591 case-control pairs in the second group, resulting in 3862 and 3913 residential addresses respectively. Of these 99% were geo-referenced.

### Conditional Logistic Regression

An investigation was conducted to look for potentially important covariates using conditional logistic regression analysis on cases and each control group. The covariates were selected based on data availability in the existing registers and existing knowledge about potential risk factors for testicular cancer and included: birth weight (kg) and birth length (cm), age of mother at birth, age of father at birth, maternal marital status (married, never married, divorced) from the Danish Birth Registry for those born after 1972, time-weighted average socioeconomic status (SES) using yearly income for 271 municipalities in Denmark, and family history of testicular cancer and of any cancer among first degree relatives with a reported primary cancer diagnosis in the Cancer Registry. Results from the logistic regression analysis were used for adjustment of key covariates in the spatial cluster analysis, as described in the following sections.

### Q-statistics background

A complete discussion on the incorporation of residential histories into disease cluster detection in the form of Q-statistics can be found elsewhere [20,23], and may be referred to for a more extensive explanation of these methods. Here we will briefly review the statistics applied to this particular study.

Q-statistics are calculated according to a nearest neighbor method. Over the course of the study period, the number of geographically nearest neighbors surrounding each individual who are a case (not a control) is calculated. A new set of Q-statistics are calculated at each time step, defined as any point in time at which at least one person changed home residences. For example, if everyone maintained the same address from 2000 to 2002 and then someone moved in 2003, the time step between calculations of the statistic would be 3 years. Each statistic is a sum over a matrix of nearest-neighbor relationships. Statistical significance is determined by permutation testing. The user specifies the number of nearest neighbors (*k)* prior to running the calculations. Each statistic is duration-weighted so as to more heavily weight individuals who have lived in the surrounding area longer. The basic formula for Q-statistics is as follows:

$$Q_{i,t}^{\left(k\right)}=c_{i}\sum_{j=1}^{k}\eta_{i,j,t}^{\left(k\right)}c_{j}$$ Equation 1

This is the count, at time *t*, of the number of *k* nearest neighbors of case *i* that are also a case. The case-control identifiers, *c*_*i*_ and *c*_*j*_,_,_ for individual *i* and *j* are binary (1 if a case, 0 if a control). When *i* is a control, $Q_{i,t}^{\left(k\right)}$= 0. The term $\eta_{i,j,t}^{\left(k\right)}$ is a binary spatial proximity matrix of nearest neighbors that is 1 when participant *j* is one of the *k* nearest neighbors of participant *i* at time *t*; and 0 otherwise. Since $Q_{i,t}^{\left(k\right)}$ is a sum of the case status of the *k* unique nearest neighbors of individual *i*, the statistic is in the range 0.*k*. When *i* is a case, low values of $Q_{i,t}^{\left(k\right)}$ indicate cluster avoidance (e.g., a case surrounded by controls), and large values indicate a cluster of cases.

Further Q-statistics build on this basic equation to identify clusters that occur according to certain spatiotemporal patterns:

$$Q_{i}^{\left(k\right)}=\int_{t=t_{0}}^{T}Q_{i,t}^{\left(k\right)}\;dt$$ Equation 2

$$Q_{t}^{\left(k\right)}=\sum_{i=1}^{n_{1}}Q_{i,t}^{\left(k\right)}$$ Equation 3

$$Q_{}^{\left(k\right)}=\sum_{i=1}^{n_{1}}Q_{i}^{\left(k\right)}$$ Equation 4

Equation 2 (the sum over all time points of Equation 1) is used to identify cases that are consistently centers of spatial clusters through time. Equation 3 represents the global statistic which indicates whether clustering occurs throughout the entire area at a particular moment in time. It is calculated by summing Equation 1 over all cases at that time point. Equation 4 further summarizes this statistic, and is the global case clustering of residential histories throughout the study area for the entire period. It is calculated by summing Equation 2 over all cases. This statistic considers all of the residential histories simultaneously for the entire study period, and is a measure of the persistence of global clustering. It is large when case clustering persists through time.

In this paper, Equation 2 is most frequently used in determining whether an individual is the center of a cluster. This is based on performance of Q-statistics as previously shown using simulations based in part on these data [23]. Equation 1 ($Q_{{}_{i,t}}^{\left(k\right)}$) is used to identify when and where an individual is the center of a local cluster. The simulation analysis [18] was undertaken to help account for multiple testing bias arising from the large number of statistical tests conducted in *Q*-statistics run across residential histories. In the simulation analysis we created clusters and evaluated the predictive capability of different versions of the local and global Q-statistics. Type 1 error was minimized and the ability to detect true clusters was maximized when we used the following criteria. First, candidate cluster constituents were identified using *p*≤0.001 for $Q_{{}_{i}}^{\left(k\right)}$ and *p*≤0.05 for $Q_{{}_{i,t}}^{\left(k\right)}$. Here we used information from the statistics defined by Equations 1 and 2 simultaneously to identify possible cluster members. Second, we required at least 4 of these cluster members to be nearest neighbors of one another in order to declare a cluster statistically significant. Equations 3 and 4 were not helpful in discerning the simulated clusters.

Statistical significance is determined by randomizing the case-control identifiers over the residential histories under the null hypothesis of no association between places of residence and case-control status. Only case-control status is randomized, maintaining the integrity of the individual residential histories, which are then used to calculate the Q statistics. The randomization procedure is repeated over many iterations to build up the distributions of the Q-statistics under the null hypothesis. For adjustment of key covariates, the null hypothesis can account for them by employing the adjusted probabilities of being a case as calculated from regression [21]. The equation for predicting the probability of being a case given the vector of covariates and risk factors for the *i* ^th^ individual is:

$$\hat{p}(c_{i}=1|x_{i})=\frac{e^{\beta 'x_{i}}}{1+e^{\beta 'x_{i}}}$$ Equation 5

Here the logit function is the natural log of the odds, and **β** is the vector of regression (slope) coefficients. Using the results of the conditional logistic regression equation, the coefficients for each variable along with values for each individual are used to assign individual probabilities of being a case in the adjusted analysis. Note that the range of possible *p*-values is determined by the number of randomizations of the null hypothesis applied. Given the computational power and time required for these analyses, 999 randomizations was the maximum reasonable number of iterations, generating a minimum *p*-value of 0.001. Given the problem of multiple testing in these and many other spatial analyses, our recent simulation analyses [23] suggested a guide for examining case-control residential histories which we implement and describe below.

### Spatial cluster analysis

Spatial cluster analyses were conducted using Q-statistics in SpaceStat (BioMedware, Inc., Ann Arbor, MI). The dataset was divided into 3 groups: all cases, seminomas, and mothers of cases and their matched controls, and each group was analyzed using different measures of time (age, calendar year, year prior to diagnosis for all cases and seminomas, and calendar year and months prior to birth for the mothers of cases). The different measures of time were used in the event that different environmental effects could be responsible for local clusters, i.e., if individuals were all located in the same region at the same date or whether they were more aligned by years prior to diagnosis (indicating similar latency period from exposure to disease manifestation). Q-statistics examine each case at each time step as a possible center of cluster, which is a thorough approach but it introduces the possibility of multiple testing. Our simulation study based in part on these data produced a guide to help account for multiple testing [23] of individual cases over time, and suggested that a possible cluster could be further evaluated if 4 or more significant cases were detected in the same area with a Q_i_^(k)^, *p* = 0.001 and Q_it_^(k)^, *p*≤0.05 using *k* = 15 nearest neighbors. We used this guide for the analyses performed in the present study. Largest clusters were re-examined at the time slice suggested by the Q-statistics using Kulldorf’s scan statistic in SaTScan (v 9.0.1) to compare with results generated by an established cluster detection method [22–25]. However, this method did not account for human mobility, thus analyses were conducted on sub-sets of the original space-time data, which included only one location per individual. These time slices were selected to match statistically significant time periods identified by Q-statistics, the approach suggested in the simulation study [23]. We used a Bernoulli model in SaTScan, and the *p*-value for test of significance was obtained from Monte Carlo simulations (999 replications). We analyzed circular clusters with a maximum cluster size of 50% of the total population.

## Results

There were 3297 cases of testicular cancer, 1871 of which were seminomas, and two independent sets of 3297 controls in this Danish population-wide case-control study. In the conditional logistic regression analyses, the only variable that was a statistically significant predictor for both seminomas and all cases using both control groups was that of having a family history of testicular cancer [23]. There were 69 cases with testicular cancer in first-degree relatives, and 39 of those were seminomas; 26 controls across both control groups with testicular cancer in first degree relatives, and 13 among controls of seminomas [9]. For all cases, the parameter estimate was 1.72 using control group 1 (*p*-value = 0.001, Hazard Ratio = 5.58) and 1.75 using control group 2 (*p*-value = 0.001, Hazard Ratio = 5.75). Given that this family history variable may reflect a common underlying exposure, there is the possibility of over-adjustment. Unadjusted spatial cluster analyses are presented, along with adjusted analyses which include the probability of being a case given a family history of testicular cancer.

The results of the unadjusted cancer cluster investigation are shown in Table 2. The table lists each test, with the number of significant individuals found and the number of individuals in the largest cluster for both control group 1 and control group 2. Also listed are number of the same individuals that were significant using both control group 1 and control group 2, and the general locations of each group of significant individuals. There was no overlap in individuals identified as centers of clusters across the two control groups in any of the analyses, including all cancer, seminomas, and mothers of cases. The largest cluster identified included 2 significant cases in the center of the cluster in Aarhus, 6–8 years prior to diagnosis, using all cases and control group 1. Another cluster was also identified in Aarhus containing residence of mothers of 2 cases in 1971, again using control group 1. All other clusters included no more than 1 significant case at the center of each cluster and persisted for many years. Given propensity for detecting false positives in this space-time cluster analysis, these results did not reach our threshold of 4 or more significant cases, which was one of the recommendations from our simulation study [23].

**Table 2:** Unadjusted Analysis.

k = 15 for all		Unadjusted				
		N significant clusters (Q_i_ = 0.001)	N persons in largest cluster	Any global stats significant?	Locations	Any matches across control groups?
**All (3297 case-control pairs)**						
**Age**						
	Group 1	4	1	No	Copenhagen, Vejle	
	Group 2	2	1	No	Copenhagen, Aarhus	No
**Calendar Year**						
	Group 1	2	1	No	Vejle, Aarhus	
	Group 2	1	1	No	Copenhagen	No
**YPD**						
	Group 1	3	2	No	Aarhus (6–8 ypd)	
	Group 2	1	1	No	Copenhagen	No
**Seminomas (1871 case-control pairs)**						
**Age**						
	Group 1	1	1	No	Copenhagen	
	Group 2	1	1	No	Copenhagen	No
**Calendar Year**						
	Group 1	0	0	No	NA	
	Group 2	0	0	No	NA	No
**YPD**						
	Group 1	2	1	No	Vejle, Skive	
	Group 2	1	1	No	Copenhagen	No
**Mothers of Cases (589 group 1/591 group 2 case-control pairs)**						
**Calendar Year**						
	Group 1	2	2	No	Aarhus (1971)	
	Group 2	0	0	No	NA	No
**Months prior to birth**						
	Group 1	0	0	No	NA	
	Group 2	0	0	No	NA	No

Results of the unadjusted cancer cluster analysis of testicular cancer in Denmark. There were *k* = 15 nearest neighbors used in every analysis. The number of significant clusters, number of persons in the largest cluster, indication of whether there were significant global statistics, the location of each cluster, and whether there were individual cases which were found in clusters using each control group are listed for each analysis. All testicular cancer cases, seminomas only, and the mothers of cases were aligned according to age at diagnosis, calendar year of diagnosis, and number of years prior to diagnosis (YPD). For the two largest clusters, the timing of the clusters is indicated.

The results from the analyses adjusted for family history of testicular cancer are shown in Table 3 and are highly similar to the unadjusted analyses. Again there was no overlap in individuals identified as centers of clusters across the two control groups in any of the analyses. There was some similarity with the unadjusted analyses in locations of significant individuals, with several of them being from either Copenhagen or Aarhus. No cluster contained more than one significant case at its center.

**Table 3:** Adjusted Analysis.

k = 15 for all		Adjusted				
		N significant clusters (Q_i_ = 0.001)	N persons in largest cluster	Any global stats significant?	Locations	Any matches across control groups?
**All (3297 case-control pairs)**						
**Age**						
	Group 1	2	1	No	Copenhagen	
	Group 2	2	1	No	Copenhagen, Vejle	No
**Calendar Year**						
	Group 1	1	1	No	Vejle	
	Group 2	1	1	No	Copenhagen	No
**YPD**						
	Group 1	2	1	No	Aarhus, Vejle	
	Group 2	3	1	No	Copenhagen	No
**Seminomas (1871 case-control pairs)**						
**Age**						
	Group 1	0	0	No	NA	
	Group 2	0	0	No	NA	No
**Calendar Year**						
	Group 1	1	1	No	Aarhus	
	Group 2	0	0	No	NA	No
**YPD**						
	Group 1	0	0	No	NA	
	Group 2	0	0	No	NA	No
**Mothers of Cases (589 group 1/591 group 2 case-control pairs)**						
**Calendar Year**						
	Group 1	1	1	No	Aarhus	
	Group 2	0	0	No	NA	No
**Months prior to birth**						
	Group 1	0	0	No	NA	
	Group 2	0	0	No	NA	No

Results of the cluster analysis of testicular cancer in Denmark, adjusted for family history of testicular cancer, following the same format as used in Table 2.

The locations of significant clusters sometimes overlapped across the different analyses. This was particularly true in the Copenhagen and Aarhus regions. However, Kulldorf’s scan statistic failed to confirm the two largest clusters in Aarhus which contained 2 significant cases at their center. Using control group 1 at 7 years prior to diagnosis, no significant clusters were found. The largest cluster detected using SaTScan contained 19 cases and was located in Copenhagen (RR = 2.0, *p* = 0.055); Q-statistics also found a possible cluster in this region of Copenhagen at *p* = 0.003 for Q_i_^(k)^, which failed to meet *p* = 0.001 that was required by our simulation study to help account for multiple testing [23]. Using maternal residential histories and control group 1 in 1971, the largest cluster detected by SaTScan was again located in Copenhagen and not statistically significant (RR = 2.02, *p* = 0.51).

## Discussion

This study used a complete record of all residential histories in Denmark to investigate local clusters of testicular cancer among residents from 1971 until diagnosis in 1991–2003. While a few small clusters were detected, no cluster contained more than two significant cases at its center, short of the four significant cases required to overcome multiple testing bias as recommended by our simulation study [23]. Further, the selection of a second control group also proved helpful to curb multiple testing bias by allowing us to examine whether the presence of a cluster remained consistent. While some clusters were found in different regions of Copenhagen using both control groups, the clusters never covered the same locations or contained the same individuals, suggesting a likelihood of being chance findings.

The results of this analysis are in line with one other small area clustering study of testicular cancer rates in England [15] which also suggested a lack of evidence of geographic clustering of testicular cancer. Our analysis goes further than this previous study by showing little evidence of local clusters even when incorporating individual-level data with changes in residency. Our method did not find evidence of local clusters in mothers’ residential histories suggesting *in utero* exposures.

There is no established protocol for detecting space-time clusters in mobile populations. When considering mobility one must consider that cases may spend different durations of time moving in and out of a cluster region. Nearest-neighbor Q-statistics allow us to investigate local and global clustering throughout residential histories in case-control studies, but are subject to chance findings resulting from multiple testing. In our simulation analyses [23] based in part on these testicular cancer data, we created many different types of clusters and arrived at a rule of thumb to help distinguish true clusters from false positives. The rule of thumb, a cluster of 4 or more individuals (Q_i_^(k)^, p = 0.001 and Q_it_^(k)^ p≤0.05) using *k* = 15, was successful for distinguishing larger clusters, which were confirmed by the scan statistic in SaTScan. We followed this approach in the analyses reported here and did not find evidence of clusters. Importantly, due to the large data set of residential histories, a single analysis took up to 12 hours. Consequently we could not explore a wide range of different levels of *k* for the analyses, but had to rely on results from the simulation study when selecting *k* = 15; although a few sensitivity analyses using *k* = 10, 20, and 100, and combining the two control groups together did not change our conclusions.

To our knowledge, this is the first examination of spatial clusters of testicular cancer using residential histories. Cases were identified in the virtually complete high-quality population-based Danish Cancer Registry [26,27], thus the study had very reliable case ascertainment. Furthermore, the Danish Civil Registration System provided an ideal frame for control selection and collection of residential addresses back to 1971 [28]. We adjusted for family history of testicular cancer as this was associated with testicular cancer and may vary spatially; however the adjustment did not change our conclusions.

The primary objective of this work was to generate insights concerning the etiology of testicular cancer, a disease that showed rapid increases in incidence throughout many Western regions in the second half of the 20^th^ century and has few established risk factors. This study design allowed us to overcome many of the limitations commonly found in spatial analyses. We used individual-level data from the Danish Cancer Registry to examine potential clusters using all cases of testicular cancer diagnosed over a 13 year period. Though it is difficult to assess the power in these analyses, we had large sample size to look for clusters only among seminomas, a more histologically similar subset of testicular cancer with a greater likelihood of common etiologic factors. We also examined potential clusters in residential history data for mothers of 589 case-control pairs and for a few years prior to pregnancy. We examined spatial patterns using multiple measures of time including locations at different ages, calendar years, and years prior to diagnosis, along with months prior to birth for the mothers.

We presented analyses both unadjusted and adjusted for family history of testicular cancer. The ability to adjust our analysis based on relevant covariates is a strength of the Q-statistics method. It is important to understand whether any detected clusters are due to variations in known covariates, or an unknown variable associated with living in a particular location. In this analysis, neither unadjusted nor adjusted results provided compelling evidence of clusters. In the adjusted results, the number of cases per potential cluster decreased suggests = ing that family history of testicular cancer may have been partially driving the results of the unadjusted analysis.

We selected two independent control groups which was helpful for interpreting the findings. Lastly, we used Q-statistics, one of the few approaches available for examining clusters throughout case-control residential histories, an approach which has been shown to have effective performance in a simulation study in this region [23]. The study could have been improved by including more years of follow-up of residential histories of cases, controls, and among mothers; the Civil Registration System began recording residential data in 1971, so it is not possible to include earlier years. Nonetheless, the study design allowed us to assess possible geographic clusters of testicular cancer using 20 years of residential histories.

The development and application of space-time cluster statistics that allow for multiple addresses and mobility is still in its infancy. Further performance evaluation of Q-statistics would help to demonstrate their utility, along with performance evaluations of other newly developed methods including the multiple address function available in SaTScan v. 9.3 (Mar 20, 2014). The limitation of not having a direct method for adjustment for multiple testing in Q-statistics was addressed in a previous simulation paper using simulated clusters, and is the reason for considering only those clusters with more than 4 individuals as significant using $Q_{{}_{i}}^{\left(k\right)}$ *p* = 0.001 and $Q_{{}_{i,t}}^{\left(k\right)}$ *p*≤0.05 [23]. Even though our null results were confirmed by time slice analysis in SaTScan, it is possible that our guideline for differentiating clusters from false positives is overly restrictive or perhaps too lenient under different situations. This is ground for future work.

Environmental influences that vary geographically either do not play a strong role in the incidence of testicular cancer in this population or our method did not detect them. The cohort effect reported in previous studies may be due to a more ubiquitous environmental factor that does not exhibit spatial variation. Additional research directions are needed to aid the pursuit of risk factors responsible for the increased risk of testicular cancer in young men.
